# Urban link travel speed dataset from a megacity road network

**DOI:** 10.1038/s41597-019-0060-3

**Published:** 2019-05-16

**Authors:** Feng Guo, Dongqing Zhang, Yucheng Dong, Zhaoxia Guo

**Affiliations:** Business School, Sichuan University, Chengdu, 610065 China

**Keywords:** Engineering, Scientific data

## Abstract

Link travel speeds in road networks are fundamental data in many research areas of traffic, transportation, and logistics. To support the research in these areas, we develop a dataset, containing the travel speeds on each road link and in different time periods together with the real road network map. The dataset is collected from a representative megacity in Western China, Chengdu. The road network of this city involves different urban road network structures. The dataset shows the realistic variations and randomness of urban link travel speeds. This enables the research of real data-driven decision-making problems in traffic, transportation and logistics areas.

## Abstract (summary)

**Table:** 

Design Type(s)	network analysis objective • modeling and simulation objective • source-based data transformation objective • time series design
Measurement Type(s)	speed
Technology Type(s)	computational modeling technique
Factor Type(s)	temporal_interval • transportation
Sample Characteristic(s)	Chengdu City Prefecture • city

Machine-accessible metadata file describing the reported data (ISA-Tab format)

## Background & Summary

With the rapid advancement of Global Positioning System (GPS) and information technology, the traffic and transportation sector is experiencing a massive increase in the amount of traffic data (e.g., vehicles’ travel trajectory data) collected. More and more real data-based studies were conducted and reported in recent years. It is reported that real data-based research could result in 30% time reduction in congestions^1^, 5% carbon emission reduction^2^, or 30% reduction in fleet size^3^ in the road network.

In the research areas of traffic, transportation and logistics, the travel speeds or travel times on road links are fundamental data for various decision-making problems, such as (a) traffic assignment^4,5^, (b) vehicle routing^2,6,7^, (c) ridesharing^8,9^, and (d) the fleet minimization problem^3^ in urban road network. These problems are generally defined in a road network, the weights of whose links are traditionally travel speeds or corresponding travel times or costs. However, to the best of the authors’ knowledge, no publicly available travel speeds dataset suitable for these decision-making problems has been reported.

Various randomnesses exist in travel speeds on real-world urban links. It has been reported that travel speeds could follow different probability distributions^10–12^, and there exist spatial and temporal correlations between travel speed on different links and in different time periods^13–15^. It is thus critical to share and publish the link travel speed dataset with real-world distributions and correlations.

On the other hand, there exist various road network structures in the real world, such as modified linear, branch, grid, 3-directional grid, 1-ring web, and 2-ring web^16^. To make the dataset more representative, it is crucial to collect the data from a city with different road network structures.

This research takes Chengdu, a megacity in Western China, as the case city, and shares the link travel speed dataset from its road network. The dataset contains the link travel speed data from June 1, 2015 to July 15, 2015 on each link and in different time periods together with the Chengdu road network map.

To obtain the link travel speed dataset, we first collect the real-time GPS trajectory data of floating vehicles in Chengdu. Then, we perform map matching to output the projected paths of the trajectories on the map and estimate the travel speeds on each link in different time periods based on the map matching results. Finally, we check the data for errors, and validate the variations and randomness of link travel speeds.

The main purpose of publishing this dataset is to facilitate real data-driven research on decision-making problems in traffic, transportation and logistics areas. Moreover, the dataset can be used in various other scenarios as well. For instance, it can be used as input data to forecast the vehicle travel speeds or travel times in urban road network^17^. The data can reflect the real traffic conditions and enable to identify the congestions^18^.

## Methods

**Fig. 1:**
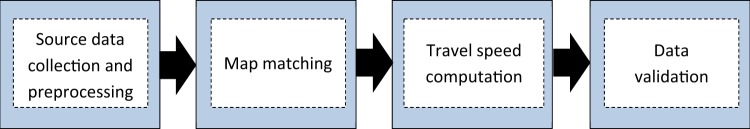
Flowchart of methodology. The figure shows the flowchart of methodology to obtain the link travel speeds in Chengdu’s road network.

Figure 1 shows the flowchart of methodology to obtain the link travel speeds in Chengdu’s road network. The steps involved are described in detail below.

### Step 1. Source data collection and preprocessing

#### Source data collection

**Fig. 2:**
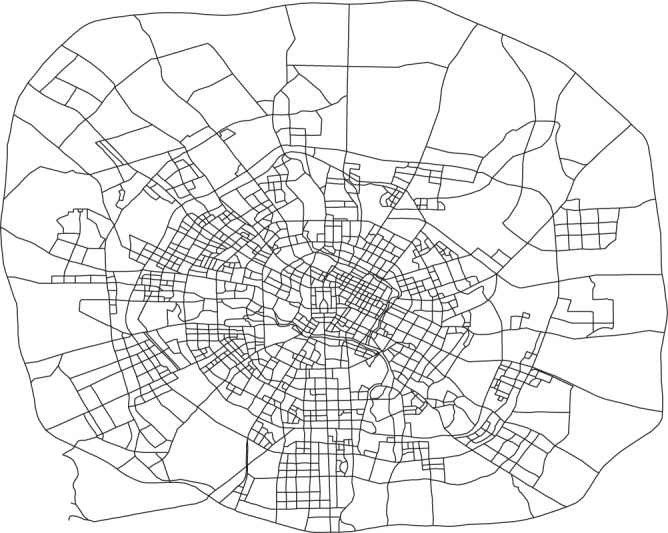
Road Network of Chengdu. The figure shows the road network of Chengdu within the ring expressway, which consists of 1,902 nodes and 5,943 directed links.

The source data for the link travel speed dataset consist of road network data and GPS trajectory data of floating vehicles. Based on OpenStreetMaps data, we use the method proposed by Karduni *et al*.^19^ to obtain the road network data, which contain the road network topology and the length of each link. The road network of Chengdu within the ring expressway is shown in Fig. 2, which consists of 1,902 nodes and 5,943 directed links. We don’t consider those links with few or no GPS trajectories in the road network. The trajectory data of floating vehicles are usually collected by the GPS-enabled devices installed in each floating vehicle during specified time intervals. This research collects the GPS trajectory data of taxis in Chengdu, China. Each trajectory sample (record) consists of the geographic location in latitude and longitude, taxi status, real-time travel speed and sampling time. All taxis use the same type of GPS-enabled devices, which ensures that the trajectory samples collected from different taxis have the same precision. The sampling rate of trajectories keeps unchanged, which is once per 10 seconds. The status of a taxi in operation switches between vacant and occupied when the taxi picks up or drops off passengers. Forty-five-day data, from 0:00 on June 1, 2015, to 23:59 on July 15, 2015, are collected. These data contain a total of 3.01 billion raw GPS trajectory samples produced by a total of more than 12,000 taxis during the data collection period.

#### Preprocessing of trajectory data

GPS signals in taxi operations could be affected or even blocked sometimes due to various electromagnetic signal shielding and interference in the city. As a result, some abnormal trajectory samples could be collected and included in the source data. It is thus crucial to preprocess the raw GPS trajectory data before map matching. On the basis of the analysis on our raw GPS trajectory data, we consider the following three types of trajectory samples as abnormal samples, and remove them from the source data.

1. Trajectory samples without key information, including taxi ID and speed values.
2. Trajectory samples with a latitude or longitude of 0°.
3. Trajectory samples having the same location information but different speed values with their proceeding trajectory points.

### Step 2. Map matching

Reliable map matching is critical to identify the accurate location of the vehicles, which outputs the projected paths of the trajectories on the map. This research performs the map matching based on the method proposed by Li *et al*.^20^. This method is selected because (1) this method is one of the most cited ones in recent years that can handle both low-frequency and high-frequency probe data of large data size well, and (2) the effectiveness of this method has been validated by comparing with the hidden Markov model-based and other map matching algorithms. The main steps are described as follows.

1. Generating the set *S* of all order-*k* links from the given Chengdu map M. Each vertex *v* in the road map has one or more order-*k* links. *k* is a given distance constant. A path is called as vertex *v*′*s* order-*k* link if it satisfies the following conditions: (a) it starts from the vertex *v*, (b) it is composed of *n* non-repeating links *l*_1_, *l*_2_, …, *l*_*n*_ such that $\sum _{i=1}^{n-1}{d}^{{l}_{i}} < k$ and $\sum _{i=1}^{n}{d}^{{l}_{i}}\ge k$, where ${d}^{{l}_{i}}$ denotes the length of link *l*_*i*_, and (c) all vertices on this path are not revisited. The larger the value of *k* is, the higher the map matching accuracy is^20^. But the number of segments increases drastically as *k* increases, which leads to the much higher computation cost. We have compared the results at different *k* and set *k* = 0.8 after considering the trade-off between map matching accuracy and computational efficiency. The map matching accuracy is 95.7% at *k* = 0.8 in terms of the accuracy criterion used in Li *et al*.’s paper^20^.
2. Joint link selection. This step assigns an order-*k* link from *S* to each trajectory sample point. Let *t* denote a travel trajectory, which is a set of *τ* trajectory sample points. We have *t* = {*a*_*t*,1_, *a*_*t*,2_, …, *a*_*t*,*τ*_} where *a*_*t*.*i*_ is the *i*^th^ trajectory sample point in trajectory *t*. The trajectory *t* is considered as a sparse and noisy sampling of an underlying path on map M. Note that the underlying path may start and end in the middle of links of M.
  We define the matching distance *d*^*m*^(*a*_*t*,*i*_, *o*) between a trajectory sample point *a*_*t*,*i*_ and an order-*k* link *o* as
  1 $${d}^{m}({a}_{t,i},o)=max\left\{{d}^{e}({a}_{t,i},o),\mathop{max}\limits_{\rho \in o}\,{d}^{e}(\rho ,t)\right\}$$
  where *d*^*e*^(*a*_*t*,*i*_, *o*) and *d*^*e*^(*ρ*, *t*) denote the Euclidean distances between the sample points (*a*_*t*,*i*_ and *ρ*) and its closest point in the piecewise linear curve (*o* and *t*). While it is straightforward to use the distance between a sample point and an order-*k* link, we add a regularizing term $\mathop{max}\limits_{\rho \in o}\,{d}^{e}(\rho ,t)$ to reflect the consistency between order-*k* link *o* and trajectory *t*. If *d*^*e*^(*a*_*t*,*i*_, *o*) is small but $\mathop{max}\limits_{\rho \in o}\,{d}^{e}(\rho ,t)$ is very large, order-*k* link *o* is likely incompatible globally to trajectory *t* because the former only indicates the local compatibility between point *a*_*t*,*i*_ and order-*k* link *o*. We allocate each trajectory sample point to the order-*k* link with the minimal matching distance.
3. Map matching using the selected order-*k* links. For each trajectory, a path is constructed from the selected order-*k* links, and take the links with trajectory sample points as this trajectory’s projected path. If two adjacent links *l*_*i*_ and *l*_*i*+1_ in the projected path are not directly connected on map M, we use the shortest path between *l*_*i*_ and *l*_*i*+1_ to connect them.

### Step 3. Travel speed computation

This step is to compute the travel speed on each link in each time period based on the map matching results, which involves 3 procedures: postprocessing of trajectory data, link travel speed estimation and speed data imputation.

#### Postprocessing of trajectory data

This procedure is to remove the trajectory samples that could decline the accuracy of travel speed estimation. We consider the following three cases.

1. Sometimes several continuous trajectory samples from a taxi have the same location and the speed value of 0 over a time period due to temporary parking. In this case, these trajectory samples are removed; otherwise, the link travel speed obtained will be smaller than the actual link travel speed.
2. The travel speeds of taxis cannot reflect the real link travel speeds during the periods of picking up or dropping off passengers. We thus find trajectory samples collected from taxis that have status switches between vacant and occupied in each period. Among the samples found, those with a speed less than the current reference speed on the link in each period are removed. That is, we only remove low-speed samples from taxis with status changes although there may be many taxis in the traffic stream that have speeds less than the reference speed. The reference speed is set as the average speed of other taxis without status changes on the same link in the same or adjacent periods.
3. The travel speeds on a link could have a very high variance in a time period due to irrational driving behaviors (e.g., speeding and stopping) or data capture errors. Speed *v* is considered as an outlier if |*v* − median(**v**)| ≥ *n* · median(**v**), where |*x*| represents the absolute value of *x*, median(**v**) represents the median of speeds of all samples on this link in the trajectory. By analysing the samples on randomly chosen 1,189 links (20% of 5,943 links), we find that setting *n* = 0.59 is able to remove outliers most effectively. This research thus takes *n* = 0.59. The corresponding trajectory sample is removed if its speed is an outlier.

#### Link travel speed estimation

The travel speeds on a link are estimated based on the location, sampling time and real-time travel speed information of trajectory sample points on the link. The sampling time indicates the time when the record is sampled, while the real-time travel speed indicates the instant travel speed of the taxi recorded by the GPS-enabled devices installed in the taxi. This research sets two minutes as one period, during which about 50,000–60,000 trajectory sample points are usually collected according to the real raw data. Thus, on average, there are about 8–10 points collected on each link in one period. In addition, the average link length is about 436.6 m in the network and the lengths of 76.7% links are less than 500 m. Considering the short-link feature, an average of 8–10 or less sample points are usually sufficient to estimate the travel speed on each link in one period. We estimate the travel speeds on a link from the following two perspectives.

On one hand, according to the method proposed by Quiroga and Bullock^21^, we estimate the travel distance of single vehicle on the link by using the real-time travel speed and sampling time information of trajectory samples, thereby get the corresponding travel speed of each vehicle on the link. We then obtain the travel speed on the link in each time period by averaging the speeds of all vehicles on the link in the same period.

Consider a link *l*. The vehicles travelled on the link are indexed by *j* (*j* = 1, …, *J*), and the trajectory sample points on the link are indexed by *k* (*k* = 1, …, *K*). Let ${p}_{j,k}^{l}$, ${t}_{j,k}^{l}$, ${v}_{j,k}^{l}$ denote the position, time, and speed of vehicle *j*′*s k*^th^ trajectory sample on link *l*, respectively.

We can calculate the travel distance ${d}_{j}^{t}$ of vehicle *j* between ${t}_{j,1}^{l}$ and ${t}_{j,K}^{l}$ as follows.

2 $${d}_{j}^{t}={\int }_{{t}_{j,1}^{l}}^{{t}_{j,K}^{l}}vdt\approx {v}_{j,1}^{l}\left(\frac{{t}_{j,2}^{l}-{t}_{j,1}^{l}}{2}\right)+\sum _{k=2}^{K-1}{v}_{j,k}^{l}\left(\frac{{t}_{j,k+1}^{l}-{t}_{j,k-1}^{l}}{2}\right)+{v}_{j,K}^{l}\left(\frac{{t}_{j,K}^{l}-{t}_{j,K-1}^{l}}{2}\right)$$

If the first and the last (*K*^th^) trajectory samples on the link are close to the two ends of the link, ${d}_{j}^{t}$ approximates the length of link *l*. Then, we can use Eqs (3) and (4) to estimate the travel speed ${v}_{j}^{l}$ of vehicle *j* and the link travel speed ${v}_{avg1}^{l}$.

3 $${v}_{j}^{l}=\frac{{d}_{j}^{t}}{{t}_{j,K}^{l}-{t}_{j,1}^{l}}$$

4 $${v}_{avg1}^{l}=\frac{1}{J}\sum _{j=1}^{J}{v}_{j}^{l}$$

The travel speed ${v}_{avg1}^{l}$ obtained above is usually smaller than the actual link travel speed. The reason is simple. The time interval for sampling two continuous trajectory points is 10 s in our source data, so the lasting time of travel speed ${v}_{j,k}^{l}$ (2 ≤ *k* ≤ *K* − 1) is 10 s as well according to Eq. (2). However, in the real world, the taxi tends to travel at the maximum speed allowed on links and low-speed travels usually last for a short time period. Therefore, the lasting time of 10 s tends to lengthen the low-speed travel time and lower down the travel speeds obtained.

On the other hand, we can first obtain the time of entering and exiting the link of each vehicle based on the location and sampling time information of trajectory samples. Then we estimate the travel speed of each vehicle ${v}_{j}^{l}$ on the link *l*, and obtain the travel speed ${v}_{avg2}^{l}$ on the link by averaging the speeds of all vehicles on the link in the same time period^21^.

Let ${v}_{j,1}^{l}$ and ${v}_{j,K}^{l}$ denote the travel speeds of vehicle *j* passing through the entry and exit ends of link *l* respectively. We use Eqs (5) and (6) to obtain the time of vehicle *j* arriving the entrance and exit ends of link *l*, ${t}_{j,ent}^{l}$ and ${t}_{j,exit}^{l}$ respectively,

5 $${t}_{j,ent}^{l}={t}_{j,1}^{l}-\frac{{d}_{j,ent}^{l}}{{v}_{j,1}^{l}}$$

6 $${t}_{j,exit}^{l}={t}_{j,K}^{l}+\frac{{d}_{j,exit}^{l}}{{v}_{j,K}^{l}}$$

where ${d}_{j,ent}^{l}$ denotes the distance between the entrance end and ${p}_{j,1}^{l}$, and ${d}_{j,exit}^{l}$ denotes the distance between ${p}_{j,K}^{l}$ and the exit end of link *l*. Then, we estimate ${v}_{j}^{l}$ and ${v}_{avg2}^{l}$ by Eqs (7) and (8).

7 $${v}_{j}^{l}=\frac{{d}^{l}}{{t}_{j,exit}^{l}-{t}_{j,ent}^{l}}$$

8 $${v}_{avg2}^{l}=\frac{1}{J}\sum _{j=1}^{J}{v}_{j}^{l}$$

where *d*^*l*^ denotes the length of link *l*. The travel speed ${v}_{avg2}^{l}$ obtained above is usually larger than the actual link travel speed. The reason is simple. According to Eqs (5) and (6), ${t}_{j,ent}^{l}$ tends to be larger and ${t}_{j,exit}^{l}$ tends to be smaller because the vehicle’s actual speeds at the ends of link are usually less than ${v}_{j,1}^{l}$ and ${v}_{j,K}^{l}$ due to the effects of traffic signals and turning vehicles. Thus, ${v}_{j}^{l}$ and ${v}_{avg2}^{l}$ tend to be larger.

To reduce the calculating deviation of travel speed values generated by the above two methods, we use Eq. (9) to compromise both values and take the final value as the link travel speed *v*^*l*^.where *w* is weight coefficient and we set *w* = 0.6 based on the analysis of a large number of speeds on urban links.

9 $${v}^{l}=w{v}_{avg1}^{l}+(1-w){v}_{avg2}^{l}$$

#### Speed data imputation

Some links cannot match with appropriate trajectory points in a specific time period in the map matching step because no trajectory samples are collected on these links in that period. As a result, we cannot obtain their travel speeds in the last procedure. Thus, the following steps are performed in turn until a valid speed value on the link is generated.

1. Obtain speed values on this link in the previous and next two periods of a current period, and take the median of these speed values as the speed on this link in the current period. This approach is called as the temporal imputation approach.
2. Obtain speed values on the immediately adjacent links (with the same direction) of this link in the same time period, and take the median of these speed values as the speed on this link in this period. This approach is called as the spatial imputation approach.
3. Obtain historical speed values on this link in the same period but in neighboring dates, and take the median of these speed values as the speed on this link in this period.

To justify the ordering of using steps 1–2, we have compared the performances of the temporal imputation approach and the spatial imputation approach. We choose out all links (1907 in total), whose travel speeds in 60 different periods are computed without using the imputation process. Then we use both approaches to generate the supplemented average speeds on these links, and compare the relative deviations of both supplemented speeds to the computed speeds. We find that the temporal approach leads to the less relative deviations for 75.6% cases. This research thus uses the temporal approach first for speed data imputation.

Of course, it is possible that there exist some approaches that work better for the data imputation on some links. We do not claim we use the best approach for data imputation, which is not the focus of this paper. Some deviations in speed estimation are inevitable and acceptable. The resulting speed values could be considered as possible real-world realizations due to the randomness and diversity of real world. After all, the dataset is reliable as long as the dataset can pass appropriate technical validation check.

### Step 4: Data validation

We perform validation steps for the link travel speed dataset obtained. Please see Section “Technical Validation” for more details.

## Data Records

The link travel speed dataset^22^ is located in figshare, which is available as 46 separate csv files described in Table 1.

**Table 1:** Data files of the dataset.

Name	Description
link.csv	Road network file.
speed_[date]_[i].csv	Travel speed files. The value of ‘[date]’ part of the filename is the real date when the travel speed data saved in the file are collected and the value of the ‘[i]’ part is 0 or 1. ‘[0]’ and [‘1] correspond to time periods 1–150 and time periods 151–300, respectively. For instance, speed_[601]_[0].csv refers to the travel speed data file for the first 150 periods on June 1, 2015. Forty-five separate speed files are included, which correspond to speed data from June 1 to July 15, 2015.

**link.csv**: This file contains the data of road network topology and the length of each link within the ring expressway of Chengdu. Relevant fields are listed out in Table 2.

**Table 2:** Summary of fields in link.csv.

Column	Data type	Description
Link	integer	Link No. of the directed link between Node_Start and Node_End.
Node_Start	integer	Node No. of the starting node of a link.
Longitude_Start	float	The longitude of the starting node.
Latitude_Start	float	The latitude of the starting node
Node_End	integer	Node No. of the end node of a link.
Longitude _End	float	The longitude of the end node.
Latitude _End	float	The latitude of the end node.
Length	float	The length of the link (unit: m).

**speed_[date]_[i].csv**: This file contains the data of link travel speeds within the ring expressway of Chengdu in different time periods of a specified date. We only obtained the link speed of 5 representative time horizons, including 3:00–5:00, 8:00–10:00, 12:00–14:00, 17:00–19:00, and 21:00–23:00. These time horizons involve rush hours, normal hours and night hours. Each time period takes 2 minutes. Thus, there are 300 time periods in total in the 5 time horizons. Relevant fields are listed out in Table 3.

**Table 3:** Summary of fields in speed_[date]_[i].csv.

Column	Data type	Description
Period	string	Time period, represented by its start and end time range.
Link	integer	Link No. of link corresponding to the file link.csv.
Speed	float	Travel speed on the link in a specific time period (unit: km/h).

## Technical Validation

This section is to validate if the link travel speed dataset can reflect the real-world link travel speeds in the road network. We validate the speed dataset by integrating numerical comparison and disciplinary analysis from the following three aspects.

### Sanity check

The first aspect of technical validation is to detect the actual errors in the link travel speed dataset. We first check that the calculations for travel speeds are inerrant. Then, we check that there are no missing or redundant speeds in the obtained dataset. Next, we check that the speed range in the dataset is valid by examining the largest speed values. We find that only 2 speed values are higher than 140 km/h in the dataset. These large speed values are valid since both of them are collected on airport expressway links during 4:00–5:00.

### Validation on variations of travel speeds

The travel speed variations are examined by observing the speeds on 100 representative links and in three different time periods, 8:00–8:02, 12:00–12:02 and 17:00–17:02. These links contain 50 downtown links located within the first ring road and 50 suburban links around the ring expressway of Chengdu. In the three time periods, the speed values (km/h) on the downtown links range within [5.15, 58.75], [6.00, 55.90] and [5.38, 56.25] respectively, while those on the suburban links range within [5.35, 107.65], [5.68, 110.45] and [7.70, 112.20] respectively. It is clear that the travel speeds on the downtown links have smaller upper limits and fluctuate within much smaller ranges than those on the suburban links. It is because there is a relatively fast and smooth traffic flow on suburban links.

### Validation on distributions and correlations of travel speeds

It has been reported that the urban link travel speeds obey the normal and lognormal distributions^12^. We fit all random speed variables (5,943 × 300 = 1,782,900 in total) with the normal and lognormal distributions using the maximum likelihood estimation method. On the basis of the one-sample Kolmogorov-Smirnov test^23^ with a significance level of 0.05, we find that 97.81% (1,743,920) of them obey the normal distributions and 1.53% (27,213) fit the lognormal distributions, whereas there are only 0.66% (11,767) random speed variables fitting neither distribution. These results are consistent with Wang *et al*.’s findings^12^.

We further examine the spatial and temporal correlation of travel speeds by calculating the Pearson correlation coefficients. Firstly, we observe the correlations of travel speeds on each link and its neighbouring links in a same time period, which are called as spatial correlations. Taking the speeds in a morning time period (8:00–8:02) as an example, we investigate the correlations of travel speeds on all links and their neighbouring links directly connected in this time period. Based on the Fisher transformation^24^ with a significance level of 0.05, we find that the speeds on 49.75% links have significant correlations with the speeds on their neighbouring links in the same direction, and the speeds on 70.68% links have insignificant correlations with the speeds on their neighbouring links in the reverse direction. Speeds in other time periods have similar results. These results are easy-to-understand, and similar correlation findings have been reported in the literature^13–15^.

**Fig. 3:**
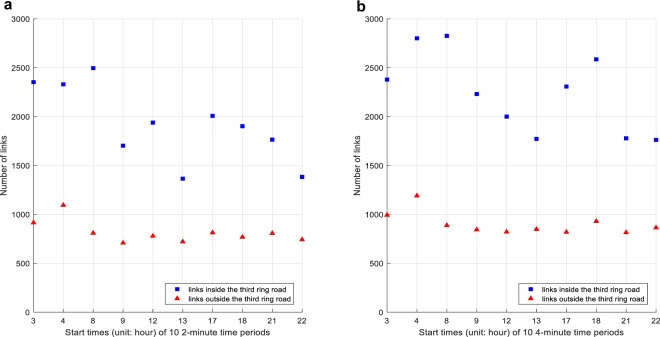
Number of links on which the temporal correlations of travel speeds are bigger than 0.5 between each of 10 time periods and its next. Subfigures **a** and **b** show the results of 2-minute and 4-minute time periods respectively. Taking subfigure **a** as an example, the two points in the longitudinal axis mean that the speeds on 2,353 links inside the third ring road and 915 links outside the third ring road have temporal correlations bigger than 0.5 between two consecutive 2-minute time periods starting at 3 am, respectively.

Next, we investigate the correlations between the travel speed on each link in each time period and the speed on the same link but in its adjacent time period, which are so-called temporal correlations. We consider 2 different time period lengths, i.e., 2 and 4 minutes, and the results are shown in Fig. 3. It can be found that, with the increase of time period length, significant temporal correlations of travel speeds on more links can be observed, especially in morning and evening rush hours. It is because the travel speeds collected from a short time period tend to exhibit intense fluctuations and noise^25^, and this would weaken the temporal correlation of the travel speeds on some links. Moreover, the number of links with strong temporal correlations outside the third ring road are less than those inside the third ring road in all 10 time periods. It indicates that the travel speeds between two consecutive time periods exhibit the stronger temporal correlation in busy traffic areas, which is in line with Rachtan *et al*.’s findings^14^. The above observations validate the temporal correlations of travel speeds in our dataset.

## Usage Notes

Since all data files are provided as csv files, the urban link travel speeds can be analysed and processed using many pieces of software, such as Pyhton, Matlab, and R. As described in Table 1, the road network data and the travel speed data are separated into different files, thus before using the data to study some decision-making problems, these two types of files need to be integrated together according to the link numbers shown in link.csv to get the travel speeds on each link in each time period. In addition, the speed data correspond to 5 representative time horizons, including 3:00–5:00, 8:00–10:00, 12:00–14:00, 17:00–19:00, and 21:00–23:00. The numbers of nodes are set to be dispersed in the road network, which could be changed to form smaller road networks.

## ISA-Tab metadata file

Download metadata file

## Data Availability

We cannot provide access to the raw source data due to their proprietary nature. As mentioned in Step 1 of the Methods section, the source data mainly contain a total of 3.01 billion GPS trajectory samples produced by more than 12,000 taxis during 45 days. As stated by Poulis *et al*.^26^, the publication of the trajectories of personal movement could lead to identity disclosure, even if directly identifying information (e.g., names of taxi drivers and passengers) is not published. Moreover, existing trajectory anonymization techniques^26,27^ cannot be used in our research because existing techniques do not care about travel speeds in trajectories and do not need the information of taxi status. However, to obtain the travel speed dataset accurately, we have to use the information of taxi status (as described in Step 3) to indicate when each taxi picks up or drops off passengers. Python (version 2.7.12) is used to produce the link travel dataset in this research. We have not shared the code because the code is dedicatedly designed for our raw source data and researchers cannot benefit from the code without the source data. Meanwhile, the code might reveal the identity of taxi drivers and raw real-time trajectory information of taxis in the road network. However, the code is straightforward, and its steps have been described in detail in the section of ‘Methods’. It is easy for a third party to exactly repeat the method.
